# Revisiting *Escherichia coli* as microbial factory for enhanced production of human serum albumin

**DOI:** 10.1186/s12934-017-0784-8

**Published:** 2017-10-05

**Authors:** Ashima Sharma, Tapan K. Chaudhuri

**Affiliations:** Kusuma School of Biological Sciences, Indian Institute of Technology Delhi, Hauz Khas, New Delhi, 110016 India

**Keywords:** Recombinant human serum albumin, *Escherichia coli*, Enhanced functional production, Cellular protein folding, Chaperones

## Abstract

**Background:**

Human serum albumin (HSA)—one of the most demanded therapeutic proteins with immense biotechnological applications—is a large multidomain protein containing 17 disulfide bonds. The current source of HSA is human blood plasma which is a limited and unsafe source. Thus, there exists an indispensable need to promote non-animal derived recombinant HSA (rHSA) production. *Escherichia coli* is one of the most convenient hosts which had contributed to the production of more than 30% of the FDA approved recombinant pharmaceuticals. It grows rapidly and reaches high cell density using inexpensive and simple subst-rates. *E. coli* derived recombinant products have more economic potential as fermentation processes are cheaper compared to the other expression hosts. The major bottleneck in exploiting *E. coli* as a host for a disulfide-rich multidomain protein is the formation of aggregates of overexpressed protein. The majority of the expressed HSA forms inclusion bodies (more than 90% of the total expressed rHSA) in the *E. coli* cytosol. Recovery of functional rHSA from inclusion bodies is not preferred because it is difficult to obtain a large multidomain disulfide bond rich protein like rHSA in its functional native form. Purification is tedious, time-consuming, laborious and expensive. Because of such limitations, the *E. coli* host system was neglected for rHSA production for the past few decades despite its numerous advantages.

**Results:**

In the present work, we have exploited the capabilities of *E. coli* as a host for the enhanced functional production of rHSA (~ 60% of the total expressed rHSA in the soluble fraction). Parameters like intracellular environment, temperature, induction type, duration of induction, cell lysis conditions etc. which play an important role in enhancing the level of production of the desired protein in its native form in vivo have been optimized. We have studied the effect of assistance of different types of exogenously employed chaperone systems on the functional expression of rHSA in the *E. coli* host system. Different aspects of cell growth parameters during the production of rHSA in presence and absence of molecular chaperones in *E. coli* have also been studied.

**Conclusion:**

In the present case, we have filled in the gap in the literature by exploiting the *E. coli* host system, which is fast-growing and scalable at the low cost of fermentation, as a microbial factory for the enhancement of functional production of rHSA, a crucial protein for therapeutic and biotechnological applications.
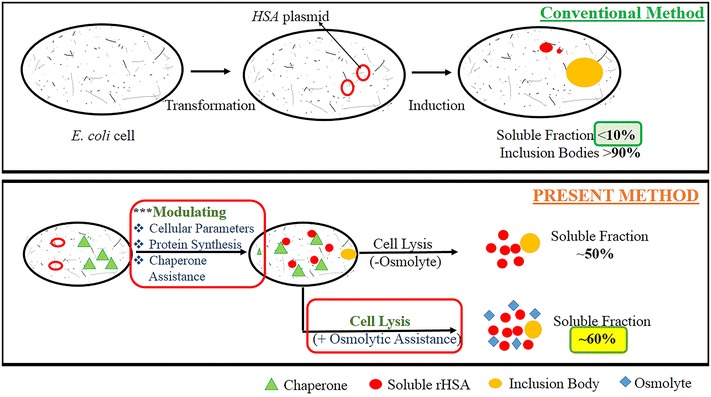

**Electronic supplementary material:**

The online version of this article (doi:10.1186/s12934-017-0784-8) contains supplementary material, which is available to authorized users.

## Background

Human serum albumin (HSA), synthesized in the liver, is the most abundant protein in the human plasma. The human protein is a 66.5 kDa, monomeric, single-chain, non-glycosylated, heart-shaped protein made up of three homologous domains comprising 585 amino acids, including 35 cysteine residues that participate in the formation of 17 disulfide bonds [1–3]. HSA is a prime determinant of plasma colloidal oncotic pressure, a universal transport and depot protein for many endogenous and exogenous metabolites and drugs while also displaying various important enzymatic, anti-inflammatory and antioxidant activities. Clinically, HSA has been employed to treat several diseases including serious burn injuries, hemorrhagic shock, hypoproteinemia, fetal-erythroblastosis and ascites caused by cirrhosis of the liver. HSA is also utilized as an excipient for vaccines, supplement in cell culture medium, the carrier of oxygen, nanodelivery of drugs and in various others biotechnological applications [1–4].

Because of its wide applications and immense therapeutic potential, annual world demand of HSA has exceeded 500 tons [5]. Currently, HSA is obtained primarily from the fractionation of collected human blood plasma [5]. Blood is a limited and an unsafe source for the production of human therapeutics. It possesses the risk of contamination by various blood-derived pathogens, like human immunodeficiency virus (HIV), hepatitis and many not yet identified, and eventually their transmission [5]. The scientific need is to eliminate the potential risk of such contamination. There exists an indispensable requirement to promote non-animal derived low-cost alternative methods for obtaining ample quantities of pathogen-free rHSA as a primary substitute for the plasma-derived form.

The non-glycosylated and single-chain polypeptide features of HSA make it less complex than the other blood-extracted proteins like plasminogen activator and clotting factors [3, 5]. These features have encouraged several investigators to produce HSA by using recombinant DNA technology and exploiting different host systems including microbial hosts, insect systems, transgenic animals and transgenic plants. Unfortunate-ly, none of the mentioned hosts have been successfully adopted for commercial production of rHSA due to their respective limitations. Transgenic animals [6, 7] and plants [8, 9] when exploited, required high capital, time consumption and yield was too low; exploiting mammalian expression systems is expensive resulting in very low yields. Consequently, several recombinant hosts such as different yeast strains like *Saccharomyces cerevisiae* [10, 11], *Pichia pastoris* [12, 13] etc. and insect systems [14], have been used for the industrial preparation of rHSA. These also have been associated with several problems including incorrect processing, poor export, hyper-glycosylation and improper folding. Moreover, it requires intense and complicated downstream processing protocols which make it impossible to produce rHSA in a cost-effective manner.

Of all the host systems*, E. coli* is one of the most convenient hosts which had contributed to the production of more than 30% of the FDA approved recombinant pharmaceuticals [15]. *E. coli* is a genetically and physiologically well-characterized organism which grows rapidly and reaches high cell density using inexpensive and simple subst-rates. The fermentation batch turnaround number for *E. coli* culture is 300 per year, which is far greater than any of the host systems available [16]. Therefore, *E. coli* derived recombinant products have more economic potential as fermentation processes are cheaper compared to the other expression hosts available.

Surprisingly, one of the production methods had remained less explored i.e. rHSA production using *E. coli* as a host. Despite all the mentioned advantages, *E. coli* has not been successfully adopted as a host for rHSA production.

The major difficulty in exploiting *E. coli* as a host for rHSA production is aggregation of this disulfide-rich, multidomain human protein during overexpression in the *E. coli* cytoplasm. Evidence had been presented that the majority of the expressed recombinant human serum albumin (rHSA) formed aggregates (more than 90% of the total expressed rHSA) leading to inclusion body formation in the reducing environment of the *E. coli* cytosol [17, 18]. Recovery of functional protein from inclusion bodies in vitro is a difficult, time-consuming, laborious and expensive task for such a multidomain and disulfide bond rich protein. Because of such limitations, *E. coli* as a host system was neglected for rHSA production. Therefore, designing advanced strategies to obtain protein in soluble form in vivo could bypass the tedious in vitro refolding strategies.

After considering the advantages of *E. coli* as a host, the present work has targeted *E. coli* as an alternate host system for rHSA production through resolving the major issue of inclusion body formation associated with it.

In the present communication, we have demonstrated the capabilities of *E. coli* as a host for the enhanced functional production of HSA. Parameters like intracellular environment, temperature, induction type, duration of induction and cell lysis conditions etc., play an important role in enhancing the level of production of the desired protein in its native form have been optimized. We have studied the effect of different types of exogenously employed chaperone systems on the functional expression of rHSA in the *E. coli* host system. We have also examined different aspects of cell growth parameters during the production of rHSA in the presence and absence of exogenous molecular chaperones—GroEL–ES and trigger factor (TF).

Here, we are reporting the development of a simple, novel and innovative method for enhanced soluble and functional production of rHSA in *E. coli*. It has been achieved through modulation of basic yet very crucial parameters like the cellular growth, protein folding, and environmental parameters. Adoption of this optimized strategy led to an appreciable improvement and enhancement in the expression levels as well as the functional and soluble proportion of the total expressed rHSA in the cytosolic fraction of the *E. coli* host system as compared to the previous reports [17, 18]. The present developed process thus paves the way for exploiting the *E. coli* host for enhanced production of a therapeutic protein with immense potential i.e. HSA in a cost-effective manner, which as per our knowledge had not been successfully attempted earlier.

## Results

### Expression of rHSA in *E. coli*

The aim of this study was to enhance the functional and soluble expression of rHSA in *E. coli* cells which had not been attempted so far, as per our knowledge.

**Fig. 1:**
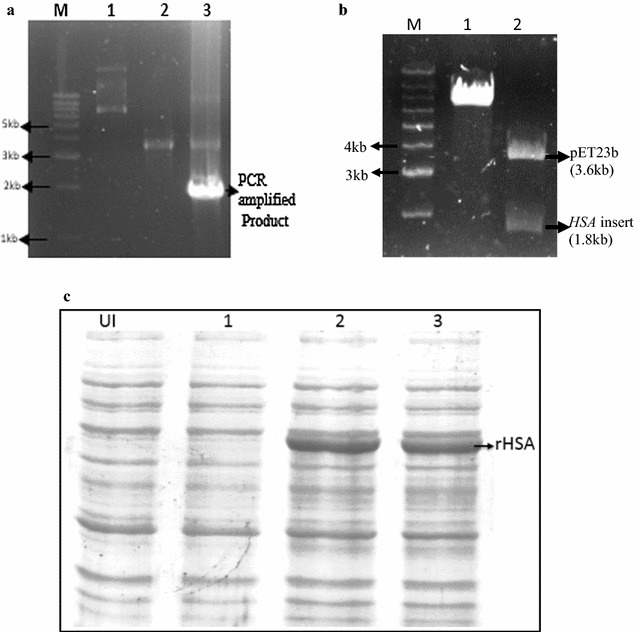
Cloning of *HSA*. **a** 1% agarose gel showing amplified PCR product and pET23b vector. Lane M, 1 kb DNA ladder; Lane 1, Uncut pET23b plasmid; Lane 2, a double digested pET23b vector with *Nhe*I and *Xho*I restriction enzymes; Lane 3, PCR amplified a product of *HSA* gene at an optimized annealing temperature of 58 °C. **b** 1% agarose gel showing the release of *HSA* gene after double digestion of constructed pETHSA vector with *Nhe*I and *Xho*I restriction enzymes. Lane M, 1 kb DNA ladder; Lane 1, Undigested pETHSA plasmid; Lane 2, digested pETHSA construct. **c** 12% SDS PAGE showing expression of rHSA in different strains of *E. coli*. Lane UI, Uninduced cells; Lane 1, Induced BL21 (DE3) *E. coli* cells; Lane 2, Induced Rosetta (DE3) *E. coli* cells; Lane 3, Induced Origami2 (DE3) *E. coli* cells

In order to achieve functional protein, the *HSA* gene was amplified by carrying out PCR from cDNA of liver cells (Fig. 1a) and was cloned into the bacterial expression vector pET23b, placing the gene under the control of the T7 promoter. The clone formation was confirmed by restriction digestion of the recombinant plasmid (pETHSA) with the respective enzymes (Fig. 1b).

To examine the expression of rHSA, the recombinant plasmid (pETHSA) was used to transform three cell lines of *E. coli*—BL21 (DE3), Rosetta (DE3) and Origami2 (DE3) followed by IPTG based induction. The cells were harvested after 8 h of post induction. SDS-PAGE analysis of the induced culture pellet revealed that a major band around 66 kDa (which is consistent with the calculated molecular mass of rHSA) was observed in the case of Rosetta (DE3) and Origami2 (DE3) cells, whereas no expression of rHSA was observed in BL21 (DE3) cells (Fig. 1c).

### Effect of process parameters on soluble and functional expression of rHSA in *E. coli*

This set of experiments were carried out to study the effect of various process parameters including cellular, folding and environmental factors on the soluble and functional expression of rHSA in *E. coli* host system. For this, recombinant cells were subjected to various conditions such as varying intracellular environment (reducing or oxidizing), varying post induction temperature (lower, moderate or high) and protein expression inducing conditions (IPTG based or autoinduction). The aim was to study and exploit an optimized set of conditions for the enhanced production of soluble and functional rHSA.

**Fig. 2:**
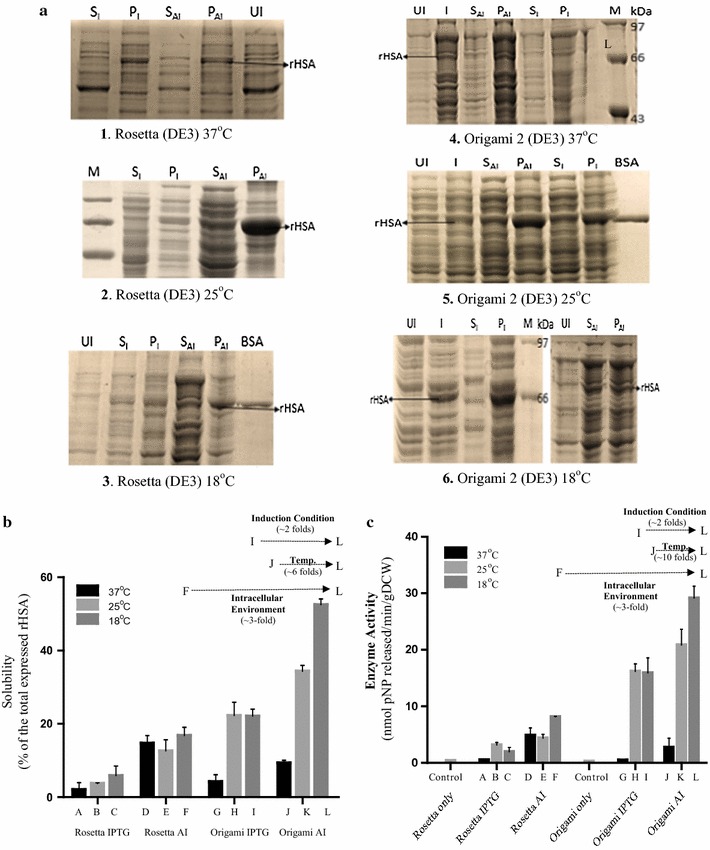
Effect of process parameters on the solubility of rHSA: densitometric analysis. **a** 12% SDS PAGE showing the change in the level of folding of rHSA subjected under different process conditions (A-L). Gel images (1–3; process conditions A–F) and (4–6; Process conditions G–L) represents the level of folding of rHSA under reducing and oxidizing intracellular environment respectively when induced via IPTG based (I) or AI and subjected to different post induction temperature. Lane UI represents uninduced cells; Lane I, induced cells; Lane S_I_, supernatant IPTG induced; P_I_, pellet IPTG induced; S_AI_, supernatant autoinduced; P_AI_, pellet autoinduced; Lane M, medium range protein molecular weight marker; Lane BSA, BSA Standard. **b** Graph shows the percentage of the soluble fraction of rHSA when expressed in *E. coli* cells subjected to different process parameters (A–L)—comprising intracellular environment, post induction temperature, and induction type- as obtained through their respective band intensities in the gel. The solubility of the rHSA has been depicted as the percentage of the rHSA obtained in the soluble fraction of the total expressed rHSA in the *E. coli* host system by carrying out densitometric analysis. Error bars represent standard error of the mean (SEM) with mean having 95% confidence limit. **c** Graph shows HSA activity units obtained when expressed in *E. coli* cells subjected to different process parameters (A–L) comprising intracellular environment (Rosetta DE3 or Origami2 DE3 *E. coli* cells), post induction temperature (18, 25, 37 °C) and induction type (IPTG or AI). Each bar represents the activity units of rHSA obtained when the normalized soluble fraction of the cell lysate, subjected to the respective condition, is analyzed for the activity assay. Rosetta only and Origami only bar represents the untransformed cells of *E. coli* strains and thus the control conditions. Here one unit of enzyme activity corresponds to one nanomole of *p*NP produced from *p*NPA per minute. Enzymatic activity expressed in the units of-nanomoles of pNP released/min/g dry cell weight. Error bars represent standard error of the mean (SEM) with mean having 95% confidence limit

To demonstrate the effect of intracellular environment on rHSA solubility and activity, Rosetta (DE3) cells and Origami2 (DE3) cells were transformed with the recombinant plasmid pETHSA. The cells were then induced with IPTG or auto-induced [19] to express rHSA in the reducing cytoplasmic environment of the Rosetta (DE3) strain or the oxidizing environment of the Origami 2 (DE3) strain of *E. coli*. The induced cells were then shifted to either of the three different temperature ranges (18, 25 or 37 °C). The cells were subsequently harvested and analyzed for rHSA solubility and activity as represented in Fig. 2 and the observations are summarized in Table 1.

**Table 1:** Combinatorial effect of various process parameters on the solubility and activity of rHSA

Process condition	Intracellular environment	Post induction temperature (°C)	Induction type	Osmolyte assistance	Solubility (% of total rHSA expressed)	Fold increase in activity
A	Reducing	37	IPTG	No	3	1
B	Reducing	25	IPTG	No	4	~ 6
C	Reducing	18	IPTG	No	6	~ 4
D	Reducing	37	Autoinduction	No	14	~ 10
E	Reducing	25	Autoinduction	No	12	~ 9
F	Reducing	18	Autoinduction	No	17	~ 16
G	Oxidizing	37	IPTG	No	4.2	~ 1
H	Oxidizing	25	IPTG	No	24.8	~ 32
I	Oxidizing	18	IPTG	No	22	~ 32
J	Oxidizing	37	Autoinduction	No	9.4	~ 5
K	Oxidizing	25	Autoinduction	No	35.7	~ 42
L	Oxidizing	18	Autoinduction	No	52.5	~ 53
M	Oxidizing	18	Autoinduction	Yes	61	~ 100

Different combinations of process parameters were used to obtain an optimized set of conditions for maximal soluble and functional expression of rHSA in *E. coli* cells.

#### Effect of intracellular environment and post-induction temperature when induced by IPTG

In order to ascertain the effect of intracellular environment on rHSA solubility and activity, recombinant *E.coli* Rosetta (DE3) cells were induced with IPTG and incubated at either 18 °C (process condition C), or 25 °C (process condition B) or at 37 °C (process condition A).

In Fig. 2 it was observed that when cells with reducing intracellular environment were induced with IPTG, under process condition A, only 3% of the total rHSA expressed could be recovered in the soluble fraction and the soluble protein activity level is designated as “1”. Under process condition B, whereby the cells under reducing intracellular environment were grown at a temperature in the range of 25 °C, 4% of the total rHSA expressed was in the soluble fraction, and there was a sixfold increase in protein activity level compared to process condition A. Under process condition C, whereby the cells were grown at a temperature in the range of 18 °C, 6% of the total rHSA expressed was in the soluble fraction, and there was a fourfold increase in protein activity level compared to process condition A.

In a similar manner, recombinant *E. coli* Origami2 (DE3) cells instead of Rosetta (DE3) cells were induced to express rHSA by IPTG mediated induction at various temperatures, namely, 18 °C (process condition I), 25 °C (process condition H), and 37 °C (process condition G) (Table 1). The results are represented in Fig. 2 and summarized in Table 1. It was observed that when cells having an oxidizing intracellular environment were cultured at 37 °C, under process condition G, only ~ 4% of the total rHSA expressed could be recovered in the soluble fraction, and the soluble protein activity level was similar to process condition A given a value of 1. Under process condition H, where Origami2 (DE3) cells were grown at 25 °C, about a sixfold increase (24.8%) soluble rHSA (of total rHSA expressed) was observed compared to process condition G. There was also a 32-fold increase in activity levels of rHSA compared to process condition G. Under process condition I, 22% of the total rHSA expressed was recovered as soluble rHSA, while a 32-fold increase in activity levels of rHSA was also observed.

#### Effect of intracellular environment and post-induction temperature when autoinduced

The cells having a reducing intracellular environment were grown at three different temperature conditions, namely 37 °C (process condition D), 25 °C (process condition E), and 18 °C (process condition F) and solubility and activity analysis were carried out as represented in Fig. 2. The observations are summarized in Table 1. Culturing of the cells at process condition D resulted in 14% of the total rHSA in the soluble fraction and the activity level was tenfold more than that observed under process condition A. Under process condition E, 12% of total expressed rHSA was soluble and the rHSA activity level was 1.5-fold more than that observed under process condition B. In process condition F, 17% of total expressed rHSA was soluble and the rHSA activity level was fourfold more than that observed under process condition C.

In a similar manner, the effect on the rHSA soluble expression in the oxidizing intracellular environment of Origami2 (DE3) under autoinduction (AI) condition was also studied as represented in Fig. 2. The observations were summarized in Table 1. Under process condition J, whereby the cells were grown at 37 °C, about 9% of total rHSA expressed was recovered in the soluble fraction. A fivefold increase in protein activity is observed compared to process condition G. In contrast, under process condition K, whereby the cells were grown at 25 °C, about 35% of the total rHSA expressed was recovered as soluble fraction, and about a 42-fold increase in protein activity was observed compared to process condition G. The cells when grown at 18 °C (process condition L), more than 50% of total rHSA expressed was obtained as soluble fraction, which showed about a 53-fold increase in protein activity compared to process condition G.

#### Effect of osmolytic assistance on soluble and functional production of rHSA

This part of the experiment deals with the demonstration of the effect of osmolytic assistance on the functional yield of the recombinant protein expressed when being extracted during the prime and crucial step of the downstream processing protocol i.e. cell lysis.

**Fig. 3:**
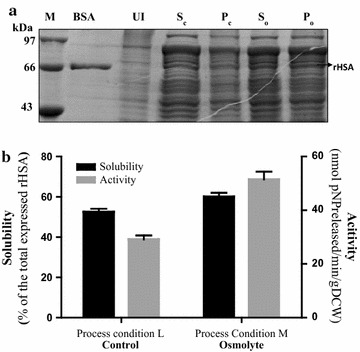
Effect of osmolytic assistance on the functional rHSA recovery during cell lysis. **a** 12% SDS PAGE showing the change in the level of folding of rHSA in the absence (Process condition L) and presence of osmolyte (Process condition M). Lane M, medium range molecular weight marker; Lane UI, Uninduced cell lysate; Lane S_c_ (supernatant control), folded rHSA in supernatant in absence of osmolyte; Lane P_c_ (pellet control), aggregated protein in the absence of osmolyte; Lane S_o_, folded rHSA in supernatant in presence of osmolyte; Lane P_o_, aggregated protein in the presence of osmolyte. **b** Graph shows the effect of osmolytic assistance during rHSA extraction on rHSA solubility and activity. The control and osmolyte panel represents the absence and presence respectively of osmolytic assistance during cell lysis for extraction of rHSA. The black bar represents the solubility of the rHSA depicted in terms of the percentage of the rHSA obtained in the soluble fraction of the total expressed rHSA. The gray bar represents the activity units obtained from carrying out the activity assay of the rHSA. One unit of enzyme activity corresponds to one nanomole of *p*NP produced from *p*NPA per minute. Enzymatic activity expressed in the units of-nanomoles of pNP released/min/g dry cell weight. Error bars represent standard error of the mean (SEM) with mean having 95% confidence limit

Autoinduced recombinant Origami2 (DE3) cells were grown at 18 °C post induction and harvested (process condition L). The harvested cells were resuspended in lysis buffer containing osmolyte (Process condition M). The cell lysate obtained after the lysis step was fractionated and analyzed for solubility and activity as represented in Fig. 3.

The effect of the osmolytic assistance during a crucial step of cell lysis can be profoundly observed as demonstrated in Fig. 3 representing the significant enhancement in the solubility ~ 10% (~ 60% of the total expressed rHSA is soluble as compared to ~ 50% without osmolytic assistance) and ~ 2-fold enhancement in the activity of rHSA was observed when was used as an osmolyte in the buffer during the cell lysis step.

### Chaperone assisted soluble and functional expression of rHSA

The present study demonstrated the effect of chaperone assistance on the expression and functional production of rHSA in *E. coli* cells. Different sets of molecular chaperone systems were employed exogenously to the rHSA expressing host in order to study and exploit the best co-expression system for the production of functional rHSA in *E. coli* cytoplasm. Recombinant molecular chaperone systems (GroEL–ES and TF) were co-expressed along with the rHSA in hosts and the effect on the level of expression, solubility, and activity of rHSA was observed and compared with the control rHSA expression system without chaperone co-expression.

#### Expression profile of rHSA in the presence and absence of chaperone system in the host

**Fig. 4:**
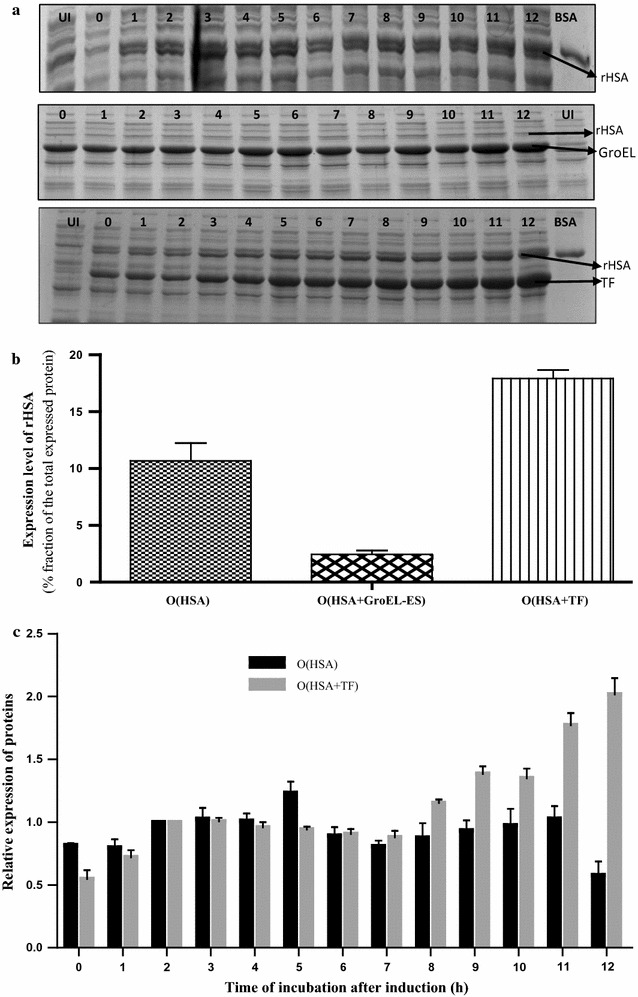
Chaperone-assisted production of rHSA in *E. coli*. A, 12% SDS PAGE showing expression level of rHSA *E. coli*—in the absence of exogenous molecular chaperones (top panel), in the presence of GroEL–ES chaperone (middle panel) and in the presence of TF chaperone (bottom panel) with different duration of incubation after induction at Post induction incubation temperature of 18 °C. Lane 1 shows Uninduced fraction, Lane 2: 0 h, Lane 3: 1 h, Lane 4: 2 h, Lane 5: 3 h, Lane 6: 4 h, Lane 7: 5 h, Lane 8: 6 h, Lane 9: 7 h, Lane 10: 8 h, Lane 11: 9 h, Lane 12: 10 h, Lane 13: 11 h, Lane 14: 12 h, Lane BSA: BSA standard (~ 66 kDa). **b** Graph shows the expression level of rHSA when coexpressed in the presence and absence of exogenously employed molecular chaperone systems in *E. coli*. The expression level is depicted in terms of percentage of rHSA expressed in *E. coli* of the total expressed protein. Error bars represent standard error of the mean (SEM) with mean having 95% confidence limit. **c** Graph shows the relative expression level of rHSA at different incubation time in the presence (represented by black bars) and absence (represented in gray bars) of TF at 18 °C as depicted through their respective band intensities in the gel. In this analysis, the protein band corresponding to the sample collected at 2 h was selected as a reference band whose value was considered as unity and the band intensities of the remaining samples were normalized against it. Error bars represent standard error of the mean (SEM) having 95% confidence limit

The expression profile of the recombinant HSA in presence and absence of molecular chaperone was estimated by SDS-PAGE analysis of the induced cell culture samples withdrawn at different time intervals (Fig. 4a). AI strategy was adopted for the expression of rHSA while GroEL–ES and TF proteins were overexpressed on induction with l-arabinose, added before the AI to ensure an abundant supply of chaperone for preventing aggregation and assisting folding of expressed recombinant protein as soon as it is produced.

Figure 4a demonstrates the expression profile of rHSA in presence and absence of chaperones. It was observed that the optimum expression level of rHSA, when expressed alone was about 10% of the total expressed protein. Whereas, the expression level of rHSA reduced to ~ 3% (fraction of the total expressed protein) when co-expressed along with the GroEL–ES. Interestingly, when co-expressed along with TF, the expression level increased to 14–20% of the total expressed protein which is about 1.5–2.0 fold more as compared to the expression of rHSA without chaperone assistance. Figure 4b graphically represents the level of expression of rHSA in presence and absence of chaperones. It was also found that the optimum rHSA expression in the absence of chaperones required 5 h of incubation after induction, whereas in presence of TF, optimum expression of rHSA required a longer induction period of 12 h as represented graphically in Fig. 4c.

#### In vivo folding of rHSA in the absence and presence of different chaperone systems

**Fig. 5:**
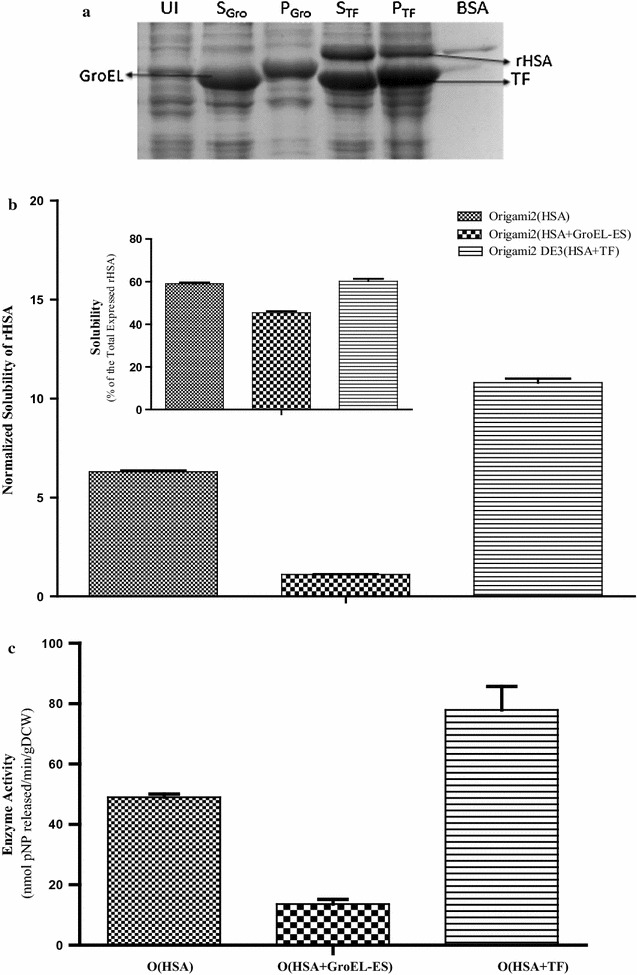
Effect of chaperone assistance on the functional production of rHSA. **a** 12%SDS PAGE showing a change in the level of folding of rHSA in presence of chaperones. Lane UI shows the uninduced cell lysate, Lane S_Gro_ (Supernatant), folded rHSA in supernatant in presence of GroEL–ES chaperone system; Lane P_Gro_ (Pellet), aggregated protein in the presence of GroEL–ES chaperone system; Lane S_TF_, folded rHSA in supernatant in presence of TF chaperone; Lane P_TF_, aggregated protein in the presence of TF chaperone. **b** Graph shows the effect of chaperone assistance on the solubility of rHSA. Normalized solubility takes into consideration the solubility with respect to the total amount of expressed rHSA. It represents the fraction percentage of the soluble expressed rHSA of the total expressed proteins in the cell lysate. The inset graph represents out of the total expressed rHSA what fraction is present in the soluble fraction (it does not take into consideration the amount of rHSA expressed). Error bars represent standard error of the mean (SEM) with mean having 95% confidence limit. **c** Graph shows the effect of chaperone assistance on the activity of rHSA. One unit of enzyme activity corresponds to one nanomole of *p*NP produced from *p*NPA per minute. Enzymatic activity expressed in the units of-nanomoles of pNP released/min/g dry cell weight. Error bars represent standard error of the mean (SEM) with mean having 95% confidence limit

The study of in vivo folding of rHSA was carried out while co-expressing in the presence of different chaperone systems–TF system and GroEL–ES system and compared to the control i.e. rHSA folding without chaperone assistance (Fig. 5). After cell lysis and successive fractionation (Process condition M, Table 1), soluble fraction appeared in the supernatant, whereas, misfolded or aggregated proteins along with the cell debris formed the pellet. Figure 5a represents the SDS-PAGE of the supernatant and pellet fraction samples of the cell lysates when rHSA was coexpressed in the presence of exogenously employed molecular chaperones GroEL–ES and TF. The extent of correctly folded functional recombinant protein was then determined by solubility and activity assay.

The inset graph plot in Fig. 5b represents the percentage of the total expressed rHSA appearing in the soluble fraction. The percentage of rHSA appearing in the soluble fraction is comparable when rHSA is expressed either in presence or absence of TF i.e. ~ 60%, whereas in presence of GroEL–ES the value decreased by ~ 15% of that of the control. Since the optimum level of expression of rHSA, when co-expressed along with different exogenous chaperone systems, is significantly variable (Fig. 4b). In order to compare the level of soluble rHSA obtained under these different expression conditions, solubility values (represented in Fig. 5b inset) were normalized by considering the variable parameter in the present case i.e. the total amount of rHSA expressed in the respective condition.

Figure 5b represents graphically the normalized solubility of rHSA when expressed in the presence and absence of chaperone systems. It was found that the solubility of rHSA increased to twofold when co-expressed along with the TF in *E. coli* host cells as compared to the control i.e. solubility of rHSA without co-expressed chaperones, whereas the solubility was diminished by sixfold when co-expressed with GroEL–ES chaperone system as compared to the control.

Furthermore, the activity assay analysis of rHSA demonstrated a marked difference in the activity of the soluble fraction’s functional content (Fig. 5c). A ~ 30% increment in the activity of soluble rHSA co-expressed along with TF was observed, whereas in the case of the GroEL–ES system the activity reduced by ~ 40% as compared to the control.

### Western blotting

Western blotting was performed to check the reactivity of the expressed rHSA towards anti-HSA antibody. To estimate accurately the effect of the developed process on the rHSA soluble production, to compare the rHSA solubility levels under the previously adopted strategy (Process condition A) and the present strategy (Process condition M) and to confirm the results discerned from the SDS-PAGE analysis, western blot was performed.

**Fig. 6:**
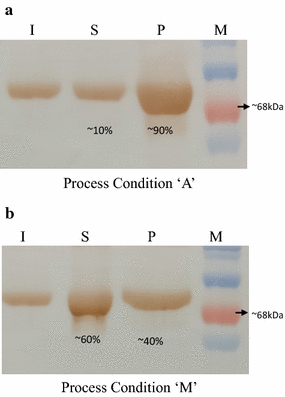
Western Blotting of rHSA. **a** western blot showing the solubility of the rHSA under process condition A (control). Lane I represents induced cells; Lane S, supernatant; Lane P, pellet; Lane M, protein molecular weight marker. **b** western blot showing the solubility of rHSA under process condition ‘M’. Lane I represents induced cells; Lane S, supernatant; Lane P, pellet; Lane M, protein molecular weight marker

Figure 6a represents the western blot of the fraction of rHSA in the soluble and insoluble fraction when expressed in the cells subjected to process condition A, the densitometric analysis was performed and results were indistinguishable to those obtained from the SDS PAGE densitometry. Under process condition A ~ 90% of the expressed rHSA is present in the pellet and ~ 10% in the soluble fraction. Similarly, Fig. 6b represents cells when subjected to process condition M, the solubility of the recombinantly expressed HSA increased to ~ 60% of the expressed rHSA as observed earlier.

### Growth studies of transformed *E. coli* cells

During the overproduction of recombinant proteins, in the present case HSA, the host cell experiences a disturbance in the intrinsic energetics which in turn directly affects its rate of growth [20]. Moreover, the production of a recombinant protein depends on the specific growth rate of the host cell. The growth profile of *E. coli* cells, when transformed with plasmids, changes to a considerable extent as compared to the wild-type strain. This set of experiments was carried out to study the effect of plasmid characteristics, induction and induction type on the growth rate of transformed Origami2 (DE3) *E. coli* cells.

**Fig. 7:**
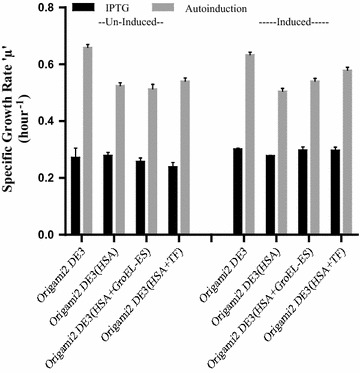
Growth studies of transformed *E. coli* cells. The graph shows the effect of plasmid characteristics, induction and induction type on the specific growth rate (µ) of the growth of Origami2 (DE3) cells. Cell growth was monitored by measurement of the turbidity at 600 nm. A plasmid containing recombinant strains were grown without induction or were subjected to induction condition (IPTG based or AI) at 37 °C and their specific growth rates were calculated as represented. The untransformed Origami2 (DE3) strain was used as a negative control in all the studies. Error bars represent standard error of the mean (SEM) with mean having 95% confidence limit

Cell growth was monitored by measurement of the turbidity at 600 nm using a spectrophotometer. Plasmids containing recombinant strains were grown without induction or were subjected to induction condition (IPTG based or AI) at 37 °C and their calculated specific growth rates are represented graphically in Fig. 7. The Origami2 (DE3) strain was used as a negative control in all the studies.

The growth rate of cells is affected by the plasmid type, number, replication, and its maintenance. It was observed through studying the growth profiles of transformed cells without induction. It was found that all the transformed cells showed a decrease in growth rate as compared to the wild-type Origami2 (DE3) cells as represented in Fig. 7 and summarized in Table 2.

**Table 2:** Specific growth rate constants (µ) for the growth of Origami2 (DE3) of *E. coli* strain under various plasmid containing situations at 37 °C

Strain	µ (h^−1^) IPTG uninduced	µ (h^−1^) IPTG induced	µ (h^−1^) AI uninduced	µ (h^−1^) AI induced
Origami2 (DE3)	0.32	0.31	0.66	0.63
Origami2 (DE3) + HSA	0.28	0.28	0.53	0.51
Origami2 (DE3){HSA + GroEL–ES}	0.26	0.29	0.52	0.54
Origami2 (DE3){HSA + TF}	0.23	0.30	0.54	0.58

It was also observed that the cells subjected to growth in different culture media showed a significant difference in their growth profile. The specific growth rate of *E. coli* cells grown in ZY medium for AI was almost twofold more than that of the cells grown in LB medium in all the cases (Table 2).

Figure 7 also represents the effect of induction on the growth rate of transformed cells. The growth rate of the transformed cells induced for expression of recombinant molecular chaperone as well as rHSA was compared with that of the uninduced transformed cells. It was observed that under uninduced conditions, strains harboring both the plasmids chaperone and HSA, showed decreased growth rate as compared to the cells harboring only HSA plasmid. However, under induced conditions, the specific growth rate of the strain carrying both the plasmids is comparable or slightly more than that of the strain carrying only one plasmid for HSA as mentioned in Table 2.

## Discussion

A range of hosts was used for functional recombinant protein production. *E. coli* is one of the preferred hosts of choice for the preparation of therapeutic proteins because of its simplicity, fast growth, and low-cost fermentation process [15, 16]. The soluble expression of functional HSA in *E. coli* is the major difficulty for investigators. In earlier attempts, rHSA was expressed in *E. coli* but the majority of the protein expressed is insoluble, in the form of inclusion bodies [17, 18]. It is known that the majority of the overexpressed proteins that have hydrophobic patches, undergo post-translational modifications or have disulfide bonds are primarily at the risk to form such amorphous aggregates [21]. HSA is a large multidomain disulfide-rich protein and it is often difficult for a disulfide bond rich protein to fold in its functional form under the normal reducing physiological environment of the *E. coli* cells.

In the present study, we have targeted and eventually resolved the prime limitation associated with the negligence of *E. coli* as a host for rHSA production. We have developed a process to overcome an unbalanced equilibrium between rHSA protein aggregation and solubilization at the cellular level by varying basic yet very crucial cellular, folding and environmental parameters. A process for the enhancement of rHSA production in *E.coli* cells has been accomplished in two phases. The first phase of the process is comprised of two tiers. The first tier of the process involves optimization of a set of cell growth parameters for enhancing soluble cytoplasmic rHSA fraction when over-expressed in the *E. coli* host. Whereas the second tier involves controlling the aggregation of soluble rHSA during cell lysis by employing the step of osmolytic assistance. In the second phase, we attempted further enhancement of functional rHSA production through the employment of chaperone-assisted folding of cellular recombinant proteins. This led to significant improvement in the cellular expression and folding of nascently synthesized rHSA polypeptide in the *E. coli* cytoplasm as discussed in detail in the subsequent sections.

It may be anticipated that the oxidizing intracellular environment of the *E. coli* host cell is preferred choice for the enhanced soluble expression of rHSA. A threefold enhancement in the solubility and activity (Fig. 2) has been attributed to the oxidizing intracellular host environment only, since for a disulfide-rich protein like HSA native disulfide bond formation is a critical factor for the proper folding of the active protein. Thus, rHSA expression in the reducing environment shows significant aggregation and misfolding as compared to its expression under oxidizing conditions. It could be deciphered from the present observation that the post-induction incubation temperature is another crucial determinant of the rHSA soluble expression in *E. coli*. The optimal functional expression of rHSA was obtained when the post induced cells were cultured at a lower temperature (Process condition L; Fig. 2). Incubation at a lower temperature renders reduced protein synthesis rates in the cells [22], thereby providing enough time for the nascent rHSA polypeptides to fold without undergoing intermolecular interaction. Also, the inclusion body formation is substantially reduced at a lower temperature and hydrophobic interaction decreases between the nascent polypeptides which bring about a reduction in the rate of aggregation [22]. Whereas at a higher temperature, the rate of aggregation is increased due to the strong temperature dependence of hydrophobic interaction [23] which makes nascent rHSA polypeptides more prone to undergo an aggregation reaction.

The induction condition also played an important role in determining the functional status of the expressed rHSA. Interestingly, about twofold enhancement in the solubility and activity (Fig. 2) was observed when a tightly regulated autoinduction strategy was adopted. It is also considered to be a highly attractive mode of induction because of the reduced requirement of monitoring the samples (no need to monitor OD_600_) and the culture reaches high cell density and helping to achieve high throughput protein expression as compared to the conventional IPTG based induction method [19]. The efforts indulged in this first tier of the process offers an optimized set of conditions which when employed together leads to a significantly enhanced soluble rHSA (~ 50% of the total rHSA expressed) production in *E. coli* cytoplasm.

It is known that a significant fraction of proteins which are not found in inclusion bodies but rather are expressed as soluble proteins in *E. coli* have a tendency to aggregate after cell lysis [24]. During extraction of soluble rHSA (~ 50% of the total expressed), further losses may occur (loss of protein due to degradation or misfolding or denaturation, loss due to increased temperature during sonication) [24], which can reduce the efficiency of production of soluble and functionally active rHSA. The presence of osmolytes during cell lysis directly impact upon the stability and solubility of proteins by assisting in protein folding and preventing protein aggregation. Trehalose is known to be one of the osmolytes which induce thermostabilization of protein’s structure and is helpful in retention of its biological activity at higher temperatures. It interacts with the side chain of proteins and contributes to their stability [25–30]. Presence of trehalose in the present case contributed to a notable enhancement of rHSA in the soluble fraction (~ 60% compared to about ~ 50% for control) and activity (~ twofold) (Fig. 3), thereby improving the recovery of the functional protein product. Hence, altogether the implementation of the first phase of the process delivers about 60% of the expressed rHSA in the soluble and functional form.

The second phase of the process targeted for further improvement in the functional production of rHSA for which we have explored assistance through chaperone co-expression. Molecular chaperones lie at the heart of protein quality control, aiding the nascent polypeptide to reach their final structure i.e. native form [31–34]. During overproduction of a recombinant protein, endogenous chaperone concentration is likely to be limited which may result in inclusion body formation. However, if chaperones are overexpressed concomitantly with the recombinant protein in vivo then they can help in the preferred folding of the expressed recombinant protein [35, 36]. Despite numerous publications reporting large quantities of soluble recombinant protein with the assistance of chaperones in *E. coli* [37–45], no report is found till date on the attempts to fold rHSA in *E. coli* cytosol with the help of co-expressed chaperones. We exploited the opportunity to introduce molecular chaperones (GroEL–ES and TF) to assess the effect on the functional preparation of rHSA in the second phase of the process.

To our surprise, it was found that the expression of rHSA increased by twofold when co-expressed along with the TF chaperone, whereas it was diminished to a negligible level when co-expressed with GroEL–ES chaperone system as compared to when rHSA was expressed without any exogenous chaperone assistance (Fig. 4). This observation could be attributed to the fact that GroEL–ES chaperone is an ATP dependent chaperone and a cell over-expressing a tetra-decameric GroEL and a heptameric GroES along with the recombinant protein of interest undergoes stress, which can limit the cellular ability for recombinant protein synthesis [46–48]. Since the amount of the total expressed rHSA in presence of GroEL–ES system was too low, it led to diminished activity values when compared to the control. TF, on the other hand, is an ATP independent chaperone which binds to the nascent polypeptide chain as soon as it is released from the ribosome into the cytosol, which reduces the aggregation of the polypeptides as well as toxicity associated with the cellular aggregates [34, 49, 50], thereby it dictates the translation speed of the rHSA and in consequence enhancing its expression yield by twofold as compared to the control. Interestingly, there are instances available in the literature where the expression of a protein increases when a cell is subjected to cold shock, because of the production of the cold shock proteins [50]. The fact that TF is a cold shock protein again strengthens the evidence of its involvement in the enhanced expression of rHSA when co-expressed in the host.

It was also discerned that the post-induction incubation time required for optimum rHSA expression in absence of chaperone was less compared to the time required in presence of the TF system. The probable reason underlying this observation is that the cells are burdened with the expression of more than one exogenously expressed recombinant proteins, leading to a reduction in the rate of protein synthesis and thereby causing longer induction period to express recombinant proteins at optimum level [22].

In the present study, it was observed that during folding of the rHSA co-expressed with chaperone TF, the percentage of soluble fraction is comparable, ~ 60% of the total expressed rHSA, with that of the soluble fraction without chaperone assistance but interestingly a substantial elevation in the functional levels of the soluble fraction by 20–30% was obtained when compared to the control (Fig. 5). This is clearly indicative of the chaperone-assisted folding of the newly translated protein. It is to be noted that TF is not enhancing the level of soluble protein through further prevention of aggregation, as the extent of the rHSA in the soluble fraction remained the same. This strongly suggests that TF has the potential to properly fold and thereby enhance the proportion of functional rHSA units in the soluble fraction of the protein when co-expressed inside the *E. coli* cytoplasm. The vast data available in the literature, on HSA folding and on TF assisted folding of proteins, when connected together helped in drawing an inference which again has supported the conclusion withdrawn from the present study i.e. TF aids in the folding of a multidomain protein like HSA. HSA has a subunit-like structure which follows a hierarchical framework model, wherein the formation of the tertiary structure occurs through a hierarchical assembly of the local elements of the tertiary structure [51, 52]. The domain folding of HSA occurs independently followed by the interdomain interactions for further folding [53]. For multidomain proteins, co-translational domain formation avoids intramolecular misfolding and is a critical factor in their folding [54]. TF, on the other hand, has been reported to allow or even accelerate productive co-translational folding processes like a domain-wise folding of multidomain proteins by serving as a folding scaffold and even facilitating a co-translational, maybe domain-wise, folding of nascent chains within its protected void [55–58]. We, from the above observations, hypothesize that TF helps in the folding of the domains of rHSA as soon as the nascent polypeptide is being synthesized. Thus, helping in the efficient and smooth folding of subsequent domains sequentially, which further assemble in a modular manner for their cooperative association in a biologically active form.

When the cell is forced to overproduce recombinant proteins, not necessarily required for its survival, it experiences an additional burden in terms of the energy utilization for the maintenance and expression of recombinant proteins [59, 60]. In the present study, we have observed that the specific growth rate of all the transformed cells was less compared to the control. It was also found that under uninduced conditions, strains harboring both the plasmids-chaperone and HSA, showed decreased growth rate as compared to the *E. coli* cells harboring only HSA plasmid (Fig. 7). However, under induced conditions, the specific growth rate of the *E. coli* strain carrying both the plasmids is comparable or slightly more than that of the strain carrying only one plasmid for HSA (Fig. 7). It may be anticipated that the availability of the abundant chaperones under induced conditions helped in the correct folding of various proteins in the cell thereby preventing their aggregation. This increases the efficiency of the cell and in turn, enhances its growth rate. Such an increase in growth rate has been reported for *E. coli* with the production of heat shock proteins [61–63]. Another significant observation was that cells subjected to growth in different culture media showed a significant difference in their growth profile (Fig. 7). The specific growth rate of *E. coli* cells grown in AI medium is almost twofold more than that of the cells grown in LB medium and induced with IPTG. It had been reported that IPTG causes a burden to the cells as IPTG after its uptake cannot be degraded inside the cell and expressing recombinant proteins through IPTG induction may slow the growth rate of the cells [64]. The AI strategy adopted here in the process takes an extra edge as lactose as an inducer is being used.

Upon succeeding with the enhanced functional rHSA production in the *E. coli* cytosol as well as the development of an improved method for protein extraction, it has become an urgent need to develop strategies for purification and its characterization, considering the fact that HSA is one of the most needed therapeutic protein with immense biotechnological applications. It is also needed to develop the strategies for the scaling up of the production for its commercial use.

The process described here for the enhanced functional production of rHSA is relatively easier and more importantly is suitable for the large-scale industrial production of the active protein. Employment of the developed strategy has significantly improved the soluble yield of the expressed rHSA in *E. coli*. Since ~ 60% of the expressed protein is soluble, which is a giant leap in production, the earlier adopted strategy of inclusion body in vitro solubilization [18] can be simply abandoned. The protein can be isolated directly from the soluble fraction of the *E. coli* cytoplasm by following the innovative steps of the currently developed process. The present method also takes into consideration the cell lysis step of the downstream processing for the enhanced recovery of soluble protein. The preliminary attempt for the purification of the rHSA through the Ni–NTA based Immobilized Metal Affinity Chromatography (IMAC) suggests 95% purity in the preparation (Additional file 1: Figure S1). Furthermore, the reactivity of the protein has been checked against anti-HSA antibody followed by preliminary ligand binding assays to assess the functionality of the purified protein, which provided positive information. Further work on the improvement of the purity level of the *E. coli* derived rHSA is in progress.

To our knowledge, this is the first report where the enhancement of the soluble and functional expression of rHSA in *E. coli* cytoplasm has been attempted successfully. The convenient and simple process developed in the present study targets and eventually resolves the major limitation of aggregation associated with the rHSA expression in *E. coli*. Thus, paving the way for the well-studied, convenient and potentially economical host *E. coli* to be exploited as an alternative host system for rHSA production.

## Conclusions

In summary, we describe a novel and innovative method for the enhanced soluble and functional production of rHSA protein in *E. coli*. To best of our knowledge, this is the first report of the successful enhancement of the soluble and functional expression of rHSA in *E. coli* cytoplasm.

The presently developed process has remarkably shortened and simplified the soluble rHSA preparation using *E. coli* as a host. The substantial reduction of rHSA aggregation in the host system has been accomplished by employment of an optimized set of crucial yet very basic cellular, environmental, folding parameters, and employment of an exogenous molecular chaperone system. The present development also takes into consideration the optimal recovery of the functional protein produced in the *E. coli* host system during the cell lysis step with cheaper ingredients and thereby providing a strategy to overcome one of the major limitations in earlier attempts of *E. coli* based rHSA production [17, 18].

The developed process thus paves the way for exploiting the most well studied, potentially low cost, scalable host system available for the enhancement of functional expression of the therapeutic protein with immense potential i.e. HSA in a cost-effective manner, which has not been attempted earlier. The present work has filled in the gap in the literature by exploiting the enhanced capabilities of *E. coli* system as an alternative host for the production of the immensely important, multi-application oriented recombinant therapeutic protein like HSA which is truly important and immensely demand driven, in a cost-effective manner.

## Methods

### Construction of fusion expression vector

The gene *HSA* (Gene ID: 213) was amplified from human liver cDNA using the primers HSA-FP (5′AAA**GCTAGC**ATGGATGCACACAAGAGTG3′, the *Nhe*I site is bold) and HSA-RP (5′GGA**CTCGAG**TAAGCCTAAGGCAGC3′; the *Xho*I site is bold).

*Nhe*I and *Xho*I restriction sites were introduced into the upstream and downstream oligonucleotide primers, respectively. PCR was performed using *Pfu* DNA polymerase (Promega, USA) with the following conditions: denaturation at 94 °C for 5 min, followed by 35 cycles of 94 °C for 30 s, 58 °C for 30 s, and 72 °C for 100 s, with a final elongation step at 72 °C for 10 min. Two blanks that contained all the reaction components except the primers or cDNA, respectively, were used as controls. The PCR fragments were double-digested with *Nhe*I and *Xho*I and then subcloned into a pET23b expression vector (Novagen, USA) that had been pre-digested with the same enzymes. The clone formation was confirmed by restriction digestion of clones with the respective enzymes. The presence of the insert in the recombinant plasmid was verified using DNA sequencing. The resulting plasmid for the expression of HSA in *E. coli* was named pETHSA. The plasmid contains the selective marker for ampicillin resistance.

### Expression of rHSA

The *E. coli* strains Origami 2 (DE3) and Rosetta (DE3) were transformed with the recombinant pET-HSA plasmid. The transformed *E. coli* cells were grown in LB medium (containing 100 µg/mL Ampicillin) till the O.D reached 0.6. The cultures were then induced with 1 mM IPTG and incubated for 12 h post induction at 37 °C with shaking at 220 rpm. A 200 μL of cell culture was aliquoted and centrifuged to collect the cell pellet. The cell pellet was resuspended in SDS gel loading buffer, boiled at 100 °C and analyzed on 12% SDS PAGE to confirm protein expression [65, 66].

### Development of *E. coli* co-expression systems

The competent *E. coli* Origami (DE3) cells were transformed with the mixture of pETHSA and respective chaperone plasmid (Takara) as mentioned in Table 3.

**Table 3:** Details of chaperone expressing plasmids

S. No.	Molecular chaperone	Plasmid	Antibiotic resistance	Inducer
1	GroEL/ES	pGro7	Chloramphenicol (20–30 µg/mL)	l-Arabinose
2	TF	pTf16	Chloramphenicol (20–30 µg/mL)	l-Arabinose

The co-transformed cells were grown in LB medium containing respective antibiotics at 37 °C with 220 rpm agitation. Cultures were induced with the chaperone and rHSA protein expression inducers. The culture was grown for 12 h post induction at 18 °C and protein expression was checked on 12% SDS PAGE.

### Determination of the relative intensities of protein bands in the SDS-PAGE gel

A Bio-Rad (USA) gel documentation unit was used for obtaining gel images and its Image-lab software was used for determining the relative quantities of protein bands present in the gel. The ‘Manual frame lanes’ toolbar from the analysis tool box was used for framing the various lanes present on the gel. The drawn lines were further adjusted to fit the size of the lanes in the gel and lane background subtraction was done to eliminate the background intensity of the gel itself from the bands. Bands were added using the ‘add band’ option and the area of the band was adjusted using the ‘adjust band’ option to optimize the region of the band to be estimated. The quantity of the selected bands was measured by selecting a band using ‘quantity tools’ from the analysis tool box. The value of intensity obtained was considered.

### Optimization of parameters for enhanced soluble expression of rHSA with- and without-chaperone assistance

The transformed *E. coli* cells were inoculated from the primary culture into a secondary culture of 100 mL of growth medium (LB medium-for IPTG based induction or AI medium) containing ampicillin (100 µg/mL) and cultured. The growth of cells was monitored and the cells were induced when the OD_600_ reached about 0.6. Protein induction was carried out either by induction using 1 mM of IPTG or by growth in AI media. Post induced cells were cultured for 12 h at three different temperature ranges (18, 25, or 37 °C). The cells were subsequently harvested and analyzed for rHSA solubility and activity.

#### Chaperone-assisted soluble expression

The co-transformed *E. coli* cells were inoculated in growth medium from the primary culture into a secondary culture of 100 mL of growth medium (LB medium-for IPTG based induction or AI medium) containing both the antibiotics-ampicillin (100 µg/mL) and chloramphenicol (20–30 µg/mL), and cultured.

The growth of cells was monitored and the cells were induced with l-Arabinose (0.2 mg/mL) when the OD_600_ reached 0.3 and was left to grow again until the OD_600_ reached 0.6. Protein induction was carried out either by induction using 1 mM of IPTG or by growth in AI media. Post induced cells were cultured for 12 h at three different temperature ranges (18, 25, or 37 °C). The cells were subsequently harvested and analyzed for rHSA solubility and activity.

### Cell lysis and fractionation

At the end of cultivation period, the normalized (for cross comparison, an equal number of cells were taken) volumes of induced cell suspension, on the basis of the number of cells per unit volume were harvested and centrifuged at 6000 rpm at 4 °C for 15–20 min. The cell pellet was resuspended in 10 mL of cold cell lysis buffer pH 7.4 (10mMTris, 100mMNaCl, and 1mMdithiothreitol) containing osmolyte and incubated in ice for 15 min. The resuspended cells were exposed to an ultrasonic cell disruptor to release the intracellular components in the lysis buffer. The sonicated cell lysate was centrifuged at 10,000 rpm for 45 min at 4 °C. The supernatant was carefully aspirated without disturbing the pellet and the pellet was resuspended in equal volume of lysis buffer.

### Determination of the solubility of rHSA: Densitometric analysis

The amount of folded protein in a cell can be estimated based on the principle that proteins with a three-dimensional structure are soluble in the cytoplasm and in aqueous buffers, whereas, denatured proteins are insoluble and occur as aggregates [43]. Thus, to estimate the extent of correct intracellular folding of rHSA, the induced cells were pelleted, resuspended in lysis buffer and lysed by sonication to release the intracellular components in the lysis buffer. The soluble components were separated from the insoluble mass by centrifugation of the cell lysate. The supernatant and the pellet were resuspended in the SDS loading buffer and analyzed by SDS-PAGE. The protein bands were visualized by staining with Coomassie Brilliant Blue R250. The relative quantity of the protein in soluble and pellet fractions was measured by selecting the whole cell extract band as reference band using the ‘quantity tools’ option of the analysis toolbox using Image Lab software in Bio-Rad Molecular Imager Gel Doc XR + unit by densitometric analysis.

$$Solubility\left( \% \right) = \frac{rHSA\,Band\,intensity \,in\,soluble\,fraction\,of\,cell\,lysate}{rHSA\,Band\,intensity\,in\,the\,whole\,cell\,lysate}*100.$$

### Activity assay of HSA

Human serum albumin exhibits esterase-like activity [67]. Esterase activity of the supernatant obtained from the fractionation cell lysate was carried out in a 1 mL reaction mixture at 25 °C containing 1 µM p-nitro phenylacetate (*p*NPA, buffered with 50 mMTris, 50mM NaCl, and 1 mM DTT, pH 7.4) as a substrate for rHSA. The formation of p-nitrophenol (*p*NP) was measured at wavelength of 410 nm spectrophotometrically after addition of normalized volume (equal number of cells per unit volume) of the cell lysate, having equal amount of total proteins estimated by Bradford’s assay, in the reaction mixture and recorded every 5 s for 10 min using the kinetics/time application in DU 800 Beckman Coulter spectrophotometer. The slope (ΔC/Δt) which represents the rate of formation of the product (p-nitrophenol) was determined from the initial linear region of the curve. An extinction coefficient (ε) of 18.3 mM^−1^cm^−1^ has been used for p-nitro phenol at 410 nm. Here, one unit activity of HSA corresponds to 1 nmol of *p*NP produced from *p*NPA per minute. HSA activity is expressed as nanomoles of *p*NP released/min/gram of dry cell weight.

$$Esterase\,Activity\,of\,HSA = \frac{{\left( {\frac{\Delta C}{\Delta t} - \frac{\Delta C}{\Delta t}blank} \right)*V_{r} *D*1000}}{{\varepsilon *V_{S*} *d}}$$

where (**ΔC/Δt**) is the slope of the activity assay curve in mM/min at 410 nm; (**ΔC/Δt blank**) is the slope of the activity assay curve in absence of enzyme in mM/min at 410 nm; **ε** is the extinction coefficient of *p*NP in mM^−1^cm^−1^; **V**_**r**_ is the reaction mixture volume in mL; **Vs** is the volume of cell lysate used in mL; **D** is the correlation between OD_600nm_ and cell dry weight in per g DCW; **d** is the path length in cm.

### Western blotting

Proteins in the cell lysate after sonication were separated by 12% SDS-PAGE under reducing conditions [65] and transferred to polyvinylidene difluoride (PVDF) membrane for 2 h at 100 V. After blocking the membrane with 5% (w/v) nonfat dry milk in phosphate-buffered saline (PBS) for 1 h, HSA primary antibodies (1:2000) were added and incubated overnight at 4 °C. After several washes with PBS containing 0.1% tween 20, appropriate HRP conjugated secondary antibodies (1:5000) were added and incubated at RT for 1 h. Blot was washed thrice and HRP activity was detected by DAB (3, 3′-Diaminobenzidine) substrate [68].

### Determination of specific growth rate constants for recombinant *E. coli* cells

Origami2 (DE3) *E. coli* cells transformed with pETHSA and cotransformed with pETHSA-pGro7, pETHSA-pTf16 were grown at 37 °C with and without induction at 220 rpm in shake flasks. At various time intervals, 1 mL of aliquots were withdrawn for turbidity measurements at 600 nm using Beckman UV-Spectrophotometer (USA).

0.3–0.6 mg/mL of Arabinose was added for the expression of a chaperone when the O.D reached 0.2–0.5; followed by induction of rHSA at 0.6–1.0 O.D with 100 µM of IPTG (in the case of IPTG based induction). AI is a self-regulated induction strategy where the inducer is already present in the growth medium [19]. To maintain non-inducing growth conditions lactose was not added in the AI medium. Expression of proteins was checked by running various withdrawn samples on 12% SDS-PAGE. *E. coli* Origami2 (DE3) strain was used as a negative control in all these studies.

For calculating the specific growth rate constant, µ, the exponential (or logarithmic) growth phase was used during which the rate of increase of cells is proportional to the number of bacteria present at that time. The specific growth rate constant µ was determined using the following equation:

$$lnN_{t} - lnN_{0} = \mu (t - t_{0} )$$

where N_t_ = number of cells at time ‘t’; N_o_ = number of cells at time ‘t = 0’; µ = specific growth rate constant; t = time in hours.

## Additional file

**Additional file 1: Figure S1.** Purification of *E. coli* derived rHSA. A, 12% SDS-PAGE showing the purified rHSA elution through Ni-NTA chromatography. Lane M, Protein molecular weight marker; Lane 1, 2, 3- Purified *E. coli* derived rHSA fractions. **B,** Western blot of the rHSA purified fractions against anti-rHSA antibody. Lane M, Protein marker; Lane1 and Lane2, Purified rHSA fractions.

## Abbreviations

AI: autoinduction
ATP: adenosine triphosphate
*E. coli*: *Escherichia coli*
HSA: human serum albumin
IPTG: isopropyl β-d-1-thiogalactopyranoside
pNP: p-nitrophenol
pNPA: p-nitro phenylacetate
rHSA: recombinant human serum albumin
TF: trigger factor
