# Supplementary figures and images for: Revisiting Escherichia coli as microbial factory for enhanced production of human serum albumin
Source: Microb Cell Fact. 2017 Oct 5;16:173. doi: 10.1186/s12934-017-0784-8 (PMC5629808; doi:10.1186/s12934-017-0784-8)

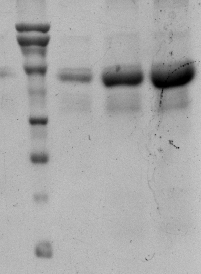


M 1 2 3

Purified rHSA

66

97

kDa

43

**A.**


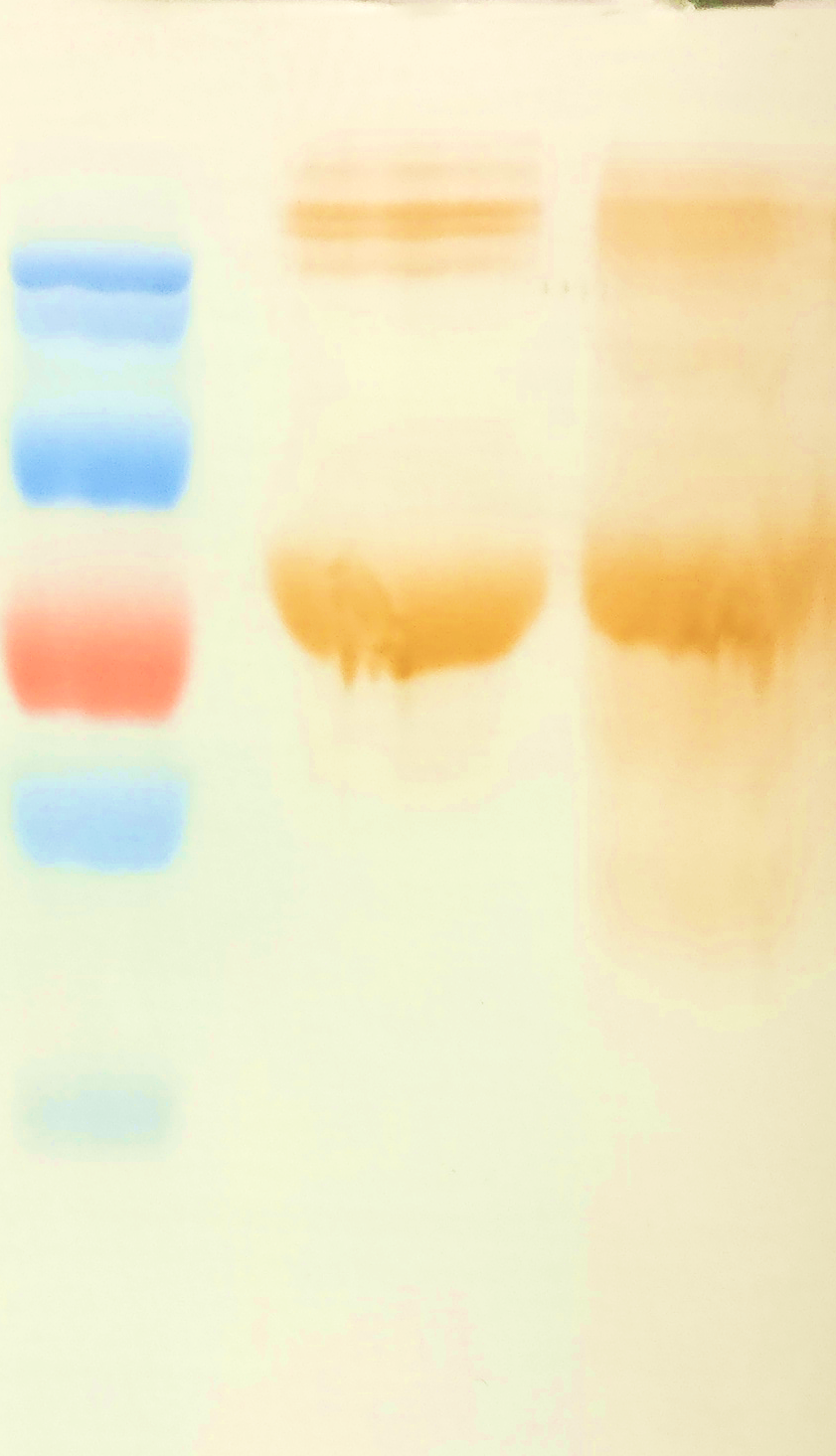


M 1 2

kDa

~68

~93

**B.**

Supplement: Supplementary file 1 — Additional file 1: Figure S1. Purification of E. coli derived rHSA. A, 12% SDS-PAGE showing the purified rHSA elution through Ni-NTA chromatography. Lane M, Protein molecular weight marker; Lane 1, 2, 3- Purified E. coli derived rHSA fractions. B, Western blot of the rHSA purified fractions against anti-rHSA antibody. Lane M, Protein marker; Lane1 and Lane2, Purified rHSA fractions. [file 12934_2017_784_MOESM1_ESM.docx]
